# Strong evidence supports the use of estradiol therapy for the treatment of vaginal inflammation: a two-way Mendelian randomization study

**DOI:** 10.1186/s40001-024-01914-4

**Published:** 2024-06-18

**Authors:** Xiaosheng Xu, Yan Liu, Weiwei Feng, Jian Shen

**Affiliations:** Department of Gynecology and Obstetrics, Ruijin Hospital Affiliated to Shanghai Jiaotong University School of Medcine, 197 Ruijiner Road, Shanghai, 200003 China

**Keywords:** Estradiol, Vaginitis, Age at menarche, *Lactobacillus*, Mendelian randomization

## Abstract

**Objective:**

Nowadays, there has been limited Mendelian randomization (MR) research focusing on the causal relationship between estradiol and vaginitis. Therefore, this study conducted a two-way MR study to clarify the causal effect and related influencing factors between them.

**Methods:**

All genetic datasets were obtained using publicly available summary statistics based on individuals of European ancestry from the IEU GWAS database. MR analysis was performed using MR-Egger, weighted median (WM) and inverse variance weighted (IVW) methods to assess the causal relationship between exposure and outcome and to validate the findings by comprehensively evaluating the effects of pleiotropic effects and outliers.

**Results:**

MR analysis revealed no significant causal relationship between estradiol and vaginitis risk. There was a negative correlation between estradiol and age at menarche (IVW, OR: 0.9996, 95% CI: 0.9992–1.0000, *P* = 0.0295; WM, OR: 0.9995, 95% CI: 0.9993–0.9998, *P* = 0.0003), and there was a positive correlation between age at menarche and vaginitis (IVW, OR: 1.5108, 95% CI: 1.1474–2.0930, *P* = 0.0043; MR-Egger, OR: 2.5575, 95% CI: 1.7664–9.6580,* P* = 0.0013). Estradiol was negatively correlated with age at menopause (IVW, OR: 0.9872, 95% CI: 0.9786–0.9959, *P* = 0.0041). However, there was no causal relationship between age at menopause and vaginitis (*P* > 0.05). In addition, HPV E7 Type 16, HPV E7 Type 18, and *Lactobacillus* had no direct causal effects on estradiol and vaginitis (*P* > 0.05). Sensitivity analyses revealed no heterogeneity and horizontal pleiotropy.

**Conclusion:**

When estrogen levels drop, it will lead to a later age of menarche, and a later age of menarche may increase the risk of vaginitis, highlighting that the longer the female reproductive tract receives estrogen stimulation, the stronger the defense ability is formed, and the prevalence of vaginitis is reduced. In conclusion, this study indirectly supports an association between reduced level of estrogen or short time of estrogen stimulation and increased risk of vaginitis.

**Supplementary Information:**

The online version contains supplementary material available at 10.1186/s40001-024-01914-4.

## Introduction

Vaginitis is caused by infection, inflammation and an imbalance of normal flora. Typical symptoms include odor, itching, irritation, burning, dysuria, or changes in vaginal discharge [1–3]. Vaginitis can negatively impact women's quality of life, with some women reporting anxiety, shame, and hygiene concerns, especially those with recurrent symptoms [4]. Hypoestrogen (atrophic) individuals may present with symptoms of vaginitis and benefit from hormone therapy or replacement therapy [5]. At present, local estrogen therapy is mainly used in the clinical treatment of atrophic vaginitis (AV) [6]. A meta-analysis of 10 clinical trials showed that vaginal estrogen therapy resulted in the greatest relief of symptoms of vaginal dryness and improvement of vaginal/vulvar atrophy [7]. Estradiol, the main estrogen produced by the ovaries, plays a vital role in keeping vaginal tissues lubricated and healthy [8]. However, existing studies are heterogeneous due to the diversity of detection methods and sample types. There is no consistent conclusion yet on the factors through which estradiol may affect vaginitis. Therefore, new comprehensive analyses are urgently needed to improve our understanding of the relationship between estradiol and vaginitis.

In contrast to the gut, which is colonized by a complex microbiome, the human vaginal ecosystem is associated with low microbial diversity, mainly dominated by a small number of Lactobacillus species [9, 10]. Microbial imbalances can manifest clinically as abnormal vaginal discharge with a "fishy" odor [11]. Human papovavirus (HPV) infection is common and spread by direct contact. Despite the high prevalence of both cervicovaginal HPV infection and vaginosis worldwide, their causal relationship remains unclear [12]. Menarche marks the beginning of the maturation and function of the female reproductive system, and approximately 50–80% of the age at puberty is determined by genetic factors [13]. Some studies have shown that early menarche may be related to factors such as obesity, high energy intake, and family environment, while late menarche may be related to factors such as emaciation and stress, among which estrogen also plays a key role [1, 14]. As age increases, a significant decrease in estrogen can reduce vaginal lubrication and cause vaginal atrophy. Vaginal atrophy is the most common problem of menopause, with serious social, psychological and medical consequences. According to the North American Menopause Society, 40 percent of postmenopausal women may develop AV [15].

A randomized controlled trial (RCT) should be an ideal study design to confirm the causal relationship between estradiol and vaginitis. However, conducting randomized controlled trials in reality faces difficulties. MR minimizes the effects of measurement error and directional causality. Because these instrumental variables (IVs) remain constant after conception and are not expected to be affected by potential founder effects, the MR approach overcomes some of the limitations of traditional epidemiological studies. Mendelian randomization (MR), a method that uses genetic variation to measure causal exposure relationships among disease risk variables, can remove confounding bias inherent in observational studies [16]. Clinical studies have revealed that vaginitis is affected by many factors. However, so far, few MR studies have focused on their causal relationship. To elucidate the causal direction between estradiol, vaginitis, age at menarche, age at menopause, HPV and *Lactobacillus*, a two-way MR study was performed using the genome-wide association study (GWAS) database in this study.

## Materials and methods

### Study design

**Fig. 1:**
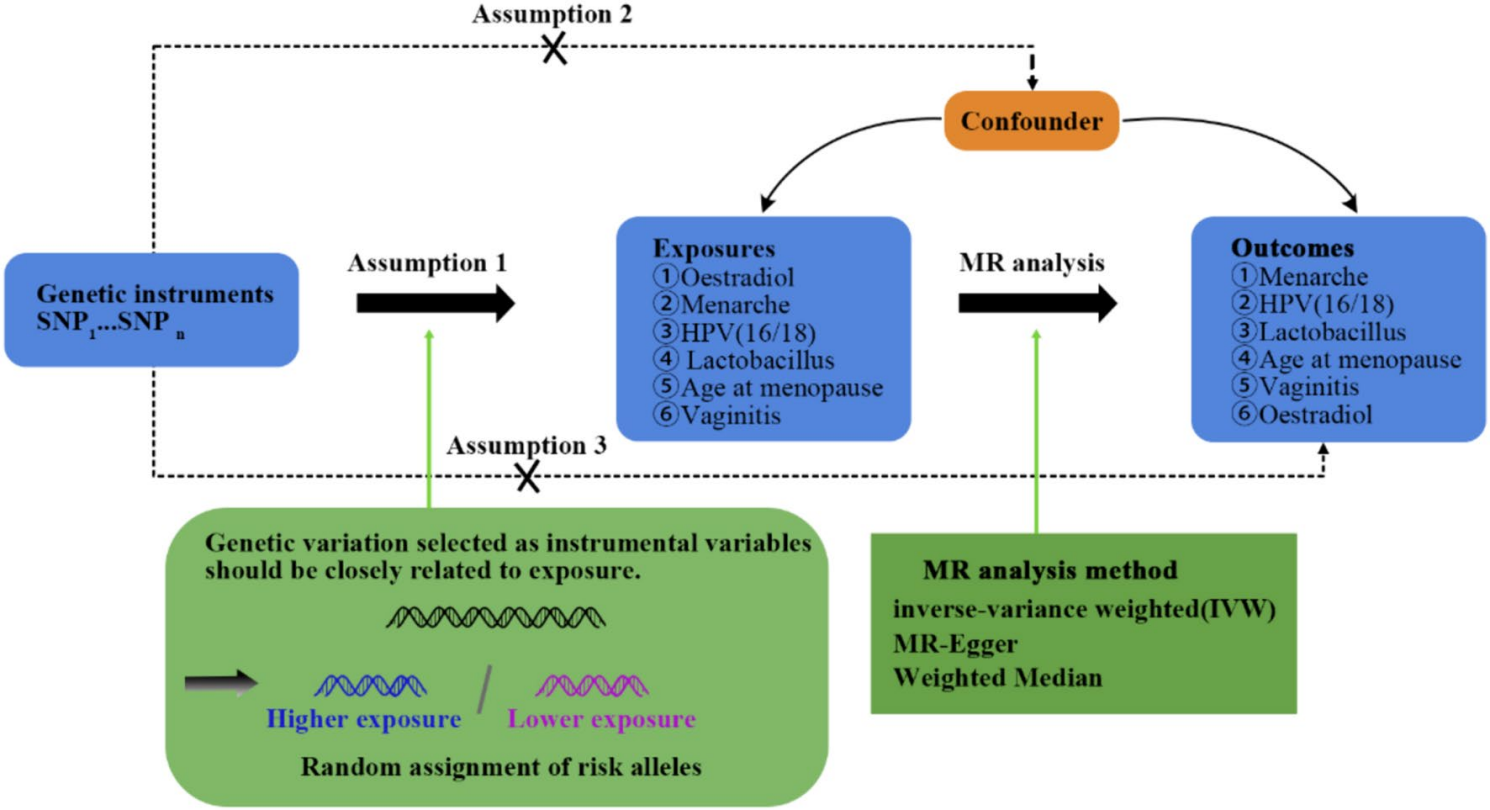
Schematic showing the steps of the three-step dual-sample MR analysis

To investigate the role of estradiol in vaginitis, we conducted Mendelian randomization (MR) analyses on two samples using GWAS data. Standard MR analysis requires that the following three model assumptions must be met [17]: (1) SNPs used as instrumental variables (IVs) are significantly associated with estradiol, vaginitis, age at menarche, age at menopause, HPV and *Lactobacillus* and reach the genome-wide significance threshold; (2) SNPs were independent of confounding factors; (3) SNPs were only associated with vaginitis through estradiol, age at menarche, age at menopause, *Lactobacillus* and HPV, but not through other pathways (Fig. 1). The first hypothesis can be tested directly using observational data. However, the last two assumptions are often difficult to test in practice. In this study, we validated our findings using the MR approach under different model assumptions.

### Data sources

The analysis in this study was based on the publicly available online database of the IEU Genome-Wide Association Studies (GWAS) (https://gwas.mrcieu.ac.uk/) [18]. Details can be found in Table 1. In addition, we provide a detailed SNP list and summary statistics from the original GWAS (Table S1).

**Table 1:** Detailed information of GWAS summary statistics used in the analysis

Phenotypes	GWAS ID	Consortium or study	Population	Sample size	Number of SNPs	Reference
Estradiol	ukb-d-30800_raw	NA	European	–	13,556,363	–
Vaginitis	finn-b-N14_VAGINITIS	NA	European	–	16,379,540	–
Menarche	ukb-a-315	Neale Lab	European	176,008	10,894,596	–
Menopause	ieu-b-4823	Within family GWAS consortium	European	4476	–	–
HPV E7 Type 16	prot-c-2623_54_4	NA	European	–	501,428	PMID:28240269
HPV E7 Type 18	prot-c-2624_31_2	NA	European	–	501,428	PMID:28240269
*Lactobacillus*	ebi-a-GCST90017030	NA	European	14,306	5,398,287	PMID:33462485

*GWAS* genome-wide association studies. *NA* not applicable

### Selection of instrumental variables

GWAS have fueled the rise of MR by identifying genetic variants that can be used as instrumental variables in a two-step framework to determine whether pending DNA methylation is present along a causal pathway between exposure and disease. In our two-sample MR, instrumental variables (IVs) were selected through the following rigorous procedure: (1) we selected SNPs that were significantly associated with exposure (*P* < 5 × 10^–8^, at the genome-wide threshold); (2) we obtained independent SNPs were identified, and the clustering threshold (*r*^2^ < 0.001, clump distance > 10,000 kb) in the PLINK clustering method was used to eliminate SNPs that caused deviations due to linkage disequilibrium (LD), and according to the allele frequency and incompatible alleles of the palindrome. Among them, low-quality SNPs were eliminated. To assess weak instrument bias, we calculated F statistics, and only SNPs with *F* < 10 were considered weak and removed [19].

### MR analysis

This study mainly used MR-Egger, weighted median (WM) and IVW (inverse variance weighted), a total of 3 algorithms for two-sample MR analysis to evaluate the causal relationship between estradiol and vaginitis. The MR-Egger approach provides a progressively consistent measure of causal effect, adjusting for horizontal pleiotropy by pooling individual SNP-specific Wald ratios via adaptive Egger regression. The WM method produces progressively consistent causal effect estimates by using the weighted median of the Wald ratios, provided that at least 50% of the variants meet the effective IV of the exclusion limits. IVW was identified as the primary method, the weighted linear regression model, in the absence of IV In the case of horizontal pleiotropy, the results have high confidence [19].

### Sensitivity analysis

The results of the primary analysis were validated by several different MR approaches with different model assumptions: (1) level multiplicity occurs when the genetic variation associated with the exposure of interest directly affects the outcome through multiple pathways other than the assumed exposure. Effectiveness: MR Egger's intercept test was used to determine the level of pleiotropy among these SNPs [20]; (2) Cochran's Q statistic of the IVW method was used to assess the heterogeneity of IV causality estimates among individuals, if the Cochran's *Q* test's *P* Values < 0.05, heterogeneity was detected. The smaller heterogeneity indicates that MR estimation is more reliable; (3) leave-one-out (LOO) analysis can be used to evaluate the influence of single SNP causal estimation, and each exposure-related SNP is sequentially discarded by LOO analysis to repeat IVW analysis. In addition, we also drew diagnostic maps to illustrate the MR results. Funnel plots can exploit the symmetry of the graphical representation to visually detect directional pleiotropy and assess the robustness of the results.

### Statistical analysis

MR is based on the principle of random distribution of genetic genes. When the frequency of SNPs is highly consistent with the changes in exposure variables, it can be preliminarily considered that SNPs are related to exposure variables. All statistical tests were two-sided and were considered to show statistical significance at a *p*-value < 0.05. The above analyses were performed using the "TwoSampleMR" package (Version: 0.5.6) and "MRPRESSO" in R software (Version: 4.1.0) " package (Version: 1.0). We first tested for heterogeneity between variant-specific estimates using Cochran Q statistic in the inverse-variance weighted model. We then explored horizontal pleiotropy via the MR-Egger and Outlier methods.

## Results

### Selected SNPs and IVs validation

The identification and information of genetic variants associated with “Estradiol”, “HPV E7 Type 16”、 “HPV E7 Type18”、 “*Lactobacillus*”、 “Age when periods started (menarche)”, “Age at menopause”, and “Vaginitis” in this study were obtained from the GWAS database. In view of the GWAS data that are significantly associated with the above disease phenotypes, after removing the biased and low-quality SNPs caused by LD, 205、27、13、54、196、30, and 108 SNPs with each F statistic above 10 were retained as IVs (*P* < 5 × 10^–8^, *r*^2^ < 0.001). Therefore, we could conclude that weak instrument bias would not substantially influence causal inference in our MR analysis.

### Causal relationship between HPV、*Lactobacillus* and estradiol and vaginitis

To assess the causal effect between estradiol and vaginal disease, MR analysis was used to infer a bidirectional causal relationship between the two. The results showed that there was a negative correlation trend between estradiol and vaginitis. However, the difference was not significant (Figures S1 and S2). Two-way MR analysis was performed on HPV E7 Type 16, HPV E7 Type 18, and genus Lactobacillus id.1837, respectively, with estradiol and vaginitis. Our MR results based on genetic data did not support a significant causal relationship between the above variables (Table S2).

### Causal relationship between estradiol and menarche

**Fig. 2:**
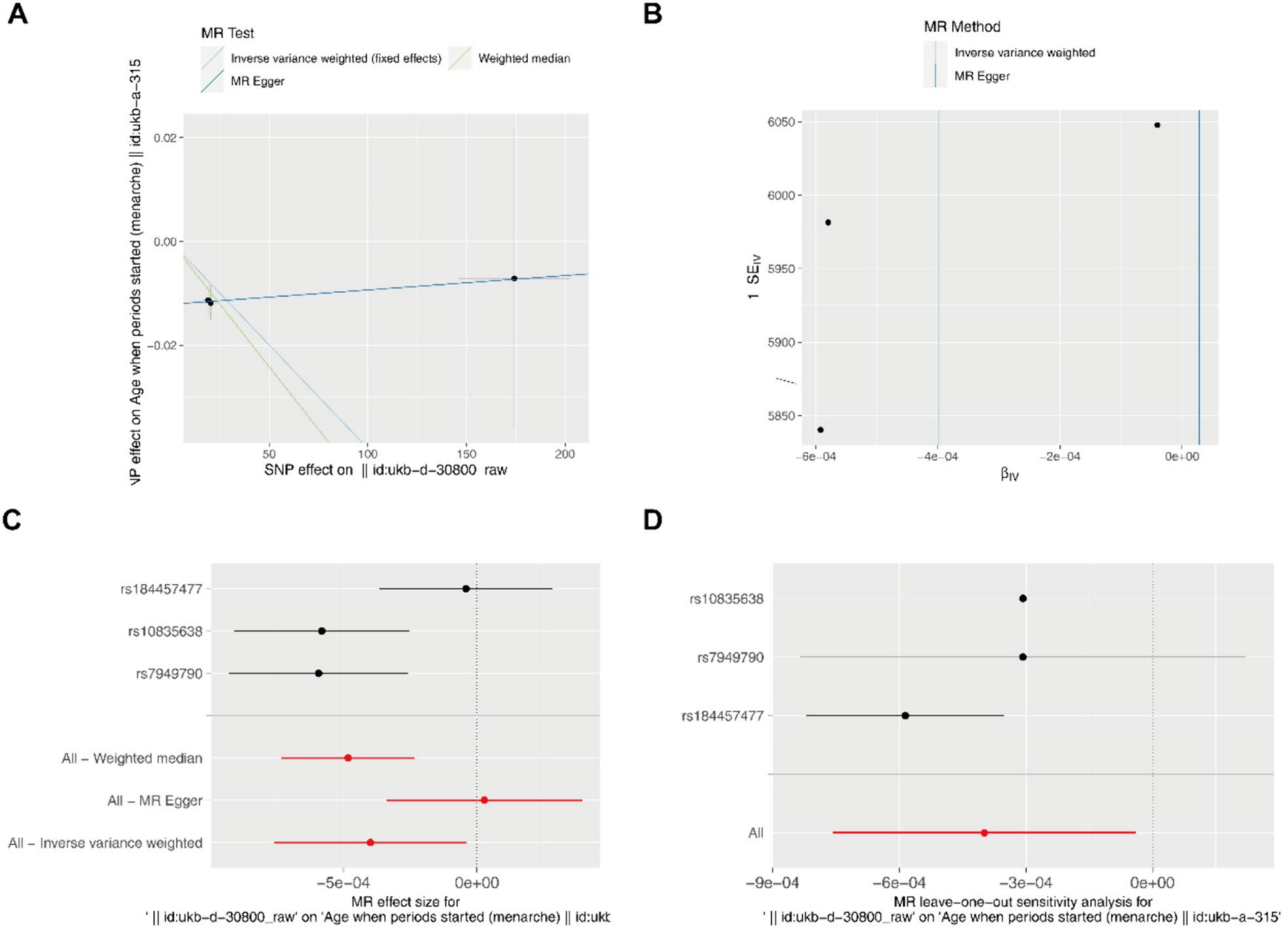
2-sample MR analysis. **A** Scatterplot of the causal effect of estradiol on age at menarche. The slope of the line indicates the magnitude of the causal relationship. **B** Funnel plot. **C** Forest plot of MR analysis representing causal estimates of estradiol on age at menarche. The circles next to each SNP indicate causal estimates for each IV, respectively, and the lowest two circles show multiple‐instrument MR analysis using Egger regression and inverse-variance weighted methods. Horizontal lines denote 95% CIs. **D** LOO analyses. The black dots represent one IVW and the red dots represent estimates using all IVs. Horizontal lines indicate 95% confidence intervals

Estrogen plays key role in early and late menarche age [1, 14]. Therefore, we used the GWAS data of age when periods started (menarche) for analysis. The results showed that the OR [95% CI] obtained by the IVW algorithm was 1.0000 [0.9992–1.0000], the β value was -0.0004, and the test *P* value was 0.0295. However, the Cochran Q test in the IVW method showed that there was some heterogeneity among the selected SNPs (*Q*_pval = 0.0283), which may be related to the small number of SNPs. The OR estimates between estradiol and age when periods started (menarche) were 1.0000 (95%CI 0.9993–0.9998; *p* = 0.0003) for the weighted median method. The MR‐Egger intercepts showed no evidence for significant directional pleiotropy (*P* intercept = 0.2283 for estradiol), suggesting that there is no directional pleiotropy in our two-sample MR. The above results show that the level of estradiol is negatively correlated with the age of menarche, and when estrogen decreases, it may lead to a delay in the age of menarche (Table 2**, **Fig. 2). In two-way analyses, the menarche-estradiol causality model was also significant, suggesting reverse causality, since the age at menarche is a continuous variable, in the MR analysis, the OR should be based on the 1SD increase of the exposure variable, so we also provide the 1SD value of the age at menarche (**Figure S3**). One standard deviation (SD) increases in age of menarche, 181,642.3 (95% CI 9.88825e−07 – 3.336679e + 16, *P* = 0.3601256) for estradiol.

**Table 2:** Causal effects of estradiol and age at menarche

Exposure	Outcome	Method	nsnp	β	se	pval	lo_ci	up_ci	or	or_lci95	or_uci95	egger_intercept	pval_intercept	*Q*	*Q*_pval
Estradiol	Age when periods started (menarche)	MR-Egger	3	2.77E−05	0.0002	0.9063	− 0.0003	0.0004	1.00002769	0.99966154	1.00039397	-0.0121	0.2283	0.0138	0.9066
		Weighted median	3	− 0.0005	0.0001	0.0003	− 0.0007	− 0.0002	0.99951788	0.99926152	0.99977432				
		Inverse variance weighted	3	− 0.0004	0.0002	0.0295	− 0.0008	− 3.98E−05	0.9996016	0.99924311	0.99996022			7.1314	0.0283

### Causal relationship between menarche and vaginitis

**Fig. 3:**
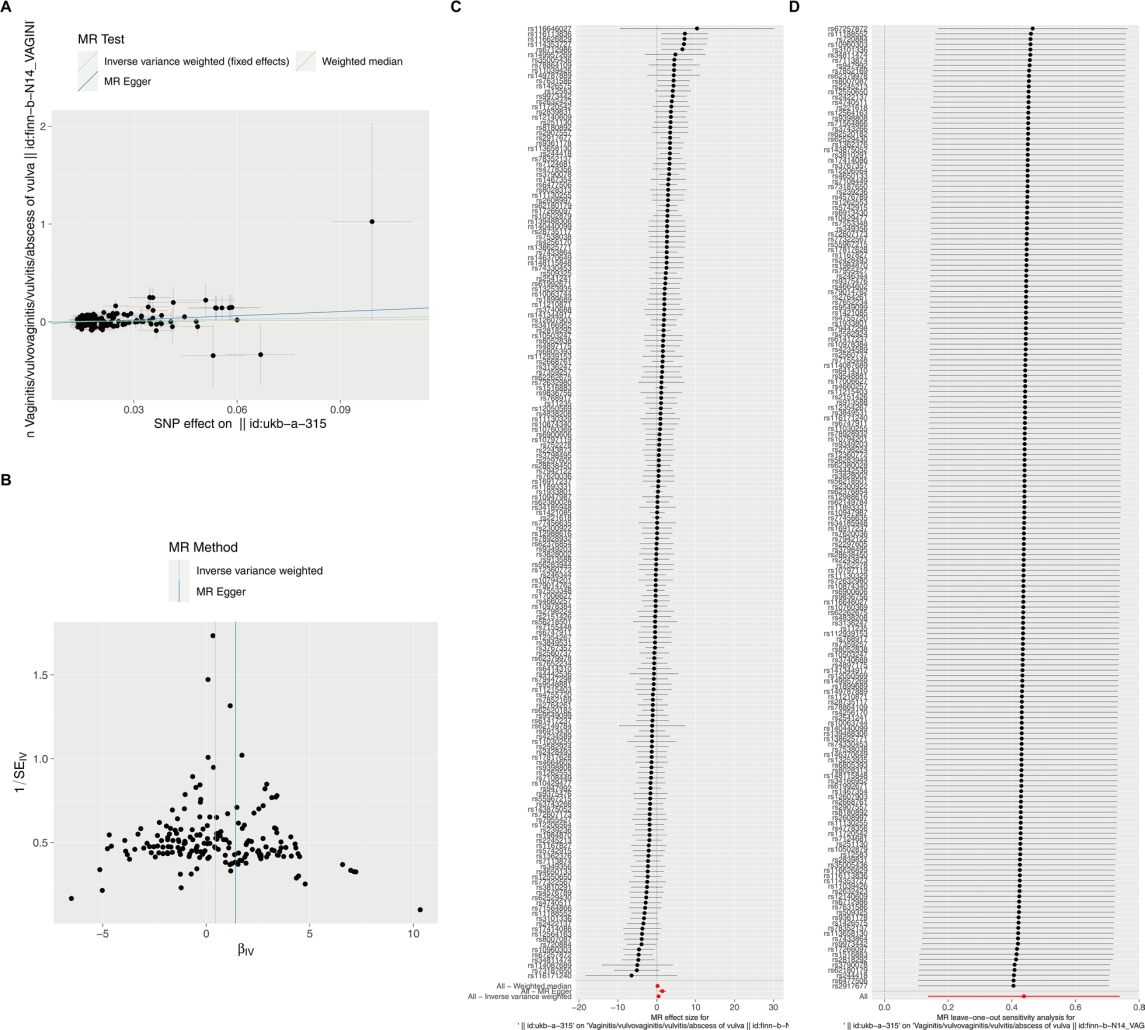
2-sample MR analysis. **A** Scatterplot of the causal effect of menarche on vaginitis. The slope of the line indicates the magnitude of the causal relationship. **B** Funnel plot. **C** Forest plot of MR analysis representing causal estimates of menarche on vaginitis. The circles next to each SNP indicate causal estimates for each IV, respectively, and the lowest two circles show multiple-instrument MR analysis using Egger regression and inverse-variance weighted methods. Horizontal lines denote 95% CIs. **D** LOO analyses. The black dots represent one IVW and the red dots represent estimates using all IVs. Horizontal lines indicate 95% confidence intervals

To assess the causal effect between menarche and vaginal disease, MR analysis was also used to infer a bidirectional causal relationship between the two. As shown in Table 3 and Fig. 3, there is a significant causal relationship between menarche and vaginal (IVW, OR: 1.5108, 95% CI: 1.1474–2.0930, *P* = 0.0043; MR-Egger, OR: 2.5575, 95% CI: 1.7664–9.6580,* P* = 0.0013). The MR‐Egger intercepts showed no evidence for significant directional pleiotropy (*P*_intercept_ = 0.1714 for menarche), suggesting that there is no directional pleiotropy in our two-sample MR. In two-way analyses, the vaginal-menarche causal model was not significant, suggesting no reverse causality (Figure S4). This evidence suggests that early stimulation of the vagina by high levels of estrogen (early menarche) may lead to the formation of a more effective barrier against external pathogens, thereby reducing the risk of vaginitis in women.

**Table 3:** Causal effects of menarche and vaginitis

Exposure	Outcome	Method	nsnp	β	se	pval	or	or_lci95	or_uci95	egger_intercept	pval_intercept	*Q*	*Q*_pval
Age when periods started (menarche)	Vaginitis	MR-Egger	183	1.4184	0.4055	0.0013	2.5575	1.7664	9.6580	− 0.0118	0.1714	231.3469	0.0068
		Weighted median	183	0.1933	0.1893	0.4054	1.1143	0.7694	1.9133				
		Inverse variance weighted	183	0.4380	0.1311	0.0043	1.5108	1.1474	2.0930			238.7962	0.0030

### Causal relationship between age at menopause and estradiol and vaginitis

**Fig. 4:**
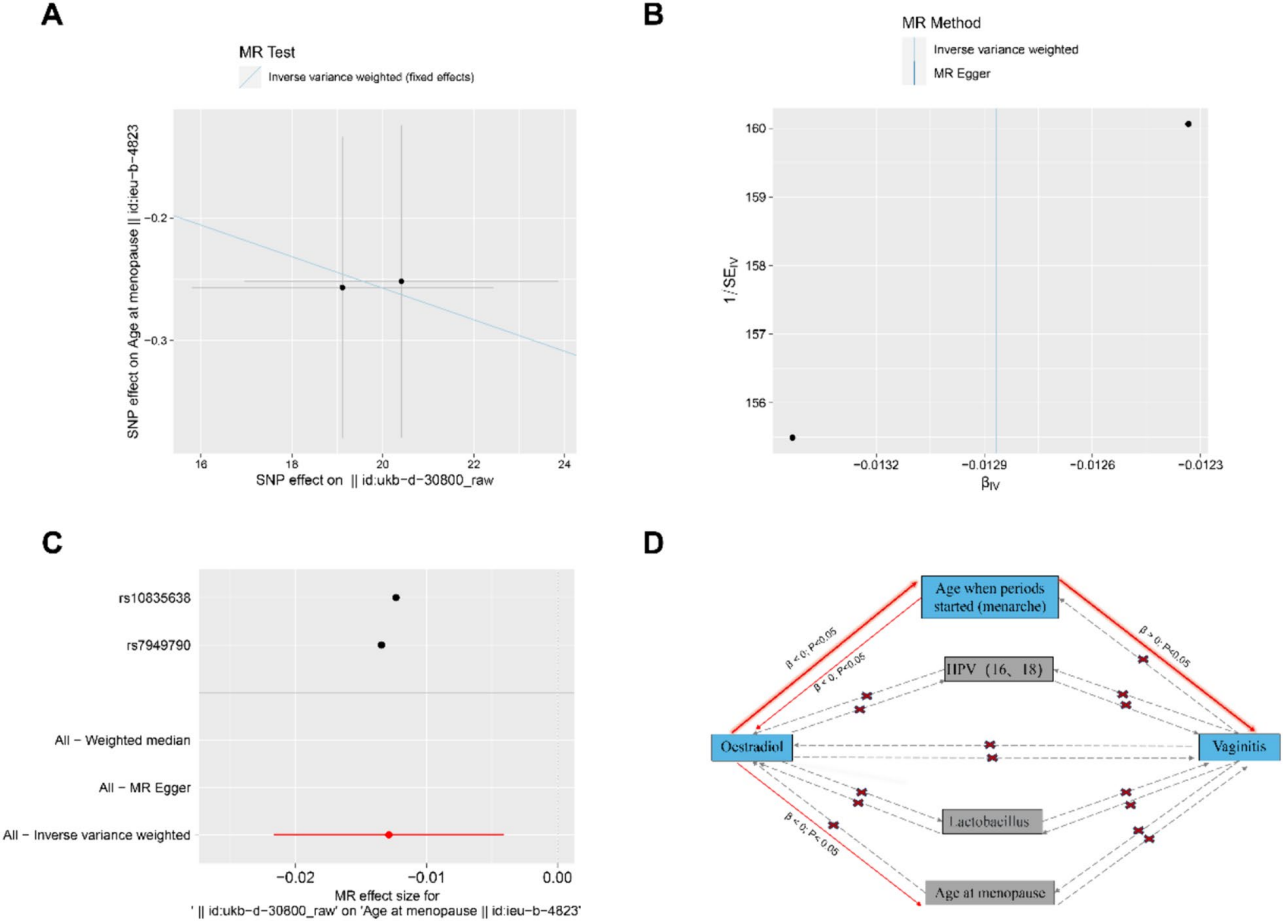
Two-sample MR analysis. **A** Scatterplot of the causal effect of estradiol on age at menopause. The slope of the line indicates the magnitude of the causal relationship. **B** Funnel plot. **C** Forest plot of MR analysis representing causal estimates of estradiol on age at menopause. The circles next to each SNP indicate causal estimates for each IV, respectively, and the lowest two circles show multiple-instrument MR analysis using Egger regression and inverse-variance weighted methods. Horizontal lines denote 95% CIs. **D** Schematic diagram of the main analysis results of this study

As age increases, a significant decrease in estrogen can lead to a decrease in vaginal lubrication, leading to vaginal atrophy, which is the most common problem during menopause [15]. Therefore, MR analysis was also performed for the causal effect between age at menopause and estradiol and vaginitis. The OR [95% CI] obtained by the IVW algorithm was 0.9872[0.9786–0.9959], the *β* value was − 0.0129, and the test *P* value was 0.0041. The Cochran Q test showed that there was no heterogeneity among the selected SNPs (*Q*_pval = 0.9024). Due to the limited number of SNPs used, the funnel chart of 2-sample MR analysis was not drawn. Inversely, increased estrogen may delay the onset of menopause (Fig. 4A–C). However, age at menopause had no significant effect on estradiol (Figure S5). Furthermore, neither age at menopause–vaginitis nor vaginitis–age at menopause causal models were significant in two-way analysis, suggesting no causal relationship between the two (Figures S6 and S7). SD increases in age of menopause, 1.001274 (95% CI 0.9689439–1.034682, *P* = 0.9394043) for vaginitis; SD increases in age of menopause, 0.9180858 (95% CI 0.05868318–12.84376, *P* = 0.9394043) for estradiol. Figure 4D shows the schematic diagram of the main analysis results of this study.

## Discussion

Atrophic vaginitis (AV) is especially common in postmenopausal women with reduced ovarian estrogen production, and hypoestrogen (atrophic) individuals may have symptoms of vaginitis [21]. Currently, a variety of estrogen therapeutic agents are available, and their safety and efficacy in the treatment of AV have been evaluated in randomized controlled clinical trials [8]. MR analysis did not provide direct evidence of a causal relationship between estradiol and vaginitis. However, there was a reciprocal causal relationship between estradiol and age at menarche, and there was also a significant causal effect between menarche and vaginitis. This result suggests that when estrogen levels drop, it will lead to a delay in the age of menarche, and a late age of menarche may increase the risk of vaginitis, which may due to the fact that the vagina is stimulated by estrogen late and does not form a strong protective barrier. In conclusion, this study indirectly supports the association between increased estrogen and decreased risk of vaginitis, which is consistent with the results of clinical trials, suggesting that vaginitis may benefit from hormone therapy or replacement therapy [5].

Menarche usually occurs between the ages of 12 and 13, as the ovaries begin to produce estrogen, and the increase in these hormones prompts the thickening and preparation of the lining of the uterus, which eventually leads to menstrual cramps [13, 22]. Age at menarche and age at natural menopause are affected by various environmental factors [23]. For age at menarche, the number of environmental covariates that could affect it was significantly smaller than age at natural menopause. For example, smoking, alcohol consumption, and duration of breastfeeding were identified as important factors affecting age at natural menopause [24]. Because they usually occur in adulthood, they probably do not affect age at menarche. However, the age of menarche is closely related to estrogen [25]. Multiple regression analysis revealed that age at menarche was inversely associated with E1 and E2 levels [26]. There are mouse data that suggest that circulating estradiol is critical for pubertal development [27]. Follicular phase serum estradiol levels are approximately twofold higher in women with early menarche compared with women with menarche at or after age 13 [28]. Estrogen excess is associated with early menstruation [29]. Although genome-wide association studies have reported associations between hundreds of genetic variants and age at menarche, studies have shown that specific genetic loci differentially influence age at menarche in healthy girls [30]. Recent genetic studies have identified rs10835638, a locus that is significantly associated with estradiol and causes menstrual cycle prolongation [31]. A later age at menarche may imply a later rise in estrogen levels, which may lead to physiological changes and poor defense against inflammation in the vaginal mucosa that make it more susceptible to infection, thereby increasing the risk of vaginitis [32, 33]. However, this relationship is complex and is influenced by the interaction of multiple factors.

Late menarche and early menopause have been shown to be associated with poor health outcomes later in life [34–36]. Menopause is an important stage in a woman's life that affects women in many ways. The clinical symptoms of menopause are the result of the gradual depletion of oocytes in the ovary, resulting in a continuous decline in estrogen levels in the body [37]. As estrogen levels decline after menopause, vaginal tissue becomes atrophic (thinner, dry, and shrunken) [38]. Ovarian estrogen production begins to decline 1 or 2 years before menopause and reaches a stable nadir about 2 years after the last menstrual period. Serum concentrations of estradiol and estrone (the major circulating estrogens) are significantly lower compared to levels in women of reproductive age [39]. Menopausal women with moderate or severe symptoms are suitable candidates for hormone therapy in the absence of contraindications or other major comorbidities [40]. We present data showing that estradiol levels are inversely correlated with age at menopause and that increased estrogen may contribute to a later age at menopause. This finding is consistent with known physiological knowledge, as estradiol is one of the important hormones in the female reproductive system and plays a key role in ovarian function and reproductive cycle regulation. Again, this result is consistent with genetic evidence that lowering the T allele (rs10835638) causes delayed menopause [31]. However, we found no significant genetic causal relationship between age at menopause and vaginitis, which means that the age of menopause itself is not directly genetically related to the occurrence of vaginitis. Vaginitis may be affected by other factors, such as environmental factors, personal lifestyle, etc. In summary, the above results show that the longer the female reproductive tract is stimulated by estrogen, the stronger the defense ability formed and the lower the prevalence of vaginitis. Therefore, understanding the relationship between estrogen and vaginal health can help develop a more personalized treatment plan that is tailored to a patient's estrogen levels and other factors.

Previous analyses have shown that *Lactobacillus* has been widely used as one of the alternatives to routine antimicrobial treatment against vaginal pathogens for the prevention of chronic vaginitis and restoration of flora balance [41, 42]. The data we presented showed that *Lactobacillus* was negatively correlated with vaginitis, but the difference was not significant. In addition, our analysis revealed an association between HPV16, HPV18 and estradiol and vaginitis, again not showing a significant causal effect. HPV16 and HPV18, as the two most common high-risk HPV subtypes, are highly associated with 70% of cervical cancers [41]. However, as far as current clinical research is concerned, the correlation between HPV and vaginitis and estradiol has not been fully explored [43].

A limitation that has to be mentioned in this study is that due to the limited number of SNPs used, there is a certain degree of heterogeneity among SNPs, which may affect our understanding of the causal effect between estrogen and vaginitis. Due to limitations of the dataset, we were unable to obtain detailed definitions of the phenotypes in each GWAS. In addition, lack of SNP data for vaginal microbes, we had to use data from gut microbes for MR analysis.

Limited by the diversity of detection methods and sample types, existing studies are heterogeneous. There is no consistent conclusion yet on the factors through which estradiol may affect vaginitis. In summary, our study provides supportive evidence for the treatment of vaginitis with estradiol from the perspective of genetic variation. While there is no direct causal relationship between estradiol and the risk of vaginitis, this study is the first to suggest that higher levels of estradiol may be linked to an earlier age of menarche. This could potentially result in a longer exposure of the vagina to estrogen, leading to the development of a stronger defense barrier and potentially reducing the risk of vaginitis. However, this process was not significantly associated with age at menopause, HPV, and *Lactobacillus*. Our study provides useful information for understanding the pathogenesis of vaginitis and provides important clues for future research directions in this field.

### Supplementary Information

Supplementary Material 1. Figure S1: 2-sample MR analysis. (A) Scatterplot of the causal effect of estradiol on vaginitis. The slope of the line indicates the magnitude of the causal relationship. (B) Forest plot of MR analysis representing causal estimates of estradiol on vaginitis. The circles next to each SNP indicate causal estimates for each IV, respectively, and the lowest two circles show multiple-instrument MR analysis using Egger regression and inverse-variance weighted methods. Horizontal lines denote 95% CIs. (C) LOO analyses. The black dots represent one IVW and the red dots represent estimates using all IVs. Horizontal lines indicate 95% confidence intervals. Figure S2: 2-sample MR analysis. (A) Scatterplot of the causal effect of vaginitis on estradiol. The slope of the line indicates the magnitude of the causal relationship. (B) Forest plot of MR analysis representing causal estimates of vaginitis on estradiol. The circles next to each SNP indicate causal estimates for each IV, respectively, and the lowest two circles show multiple-instrument MR analysis using Egger regression and inverse-variance weighted methods. Horizontal lines denote 95% CIs. (C) LOO analyses. The black dots represent one IVW and the red dots represent estimates using all IVs. Horizontal lines indicate 95% confidence intervals. Figure S3: 2-sample MR analysis. (A) Scatterplot of the causal effect of menarche on estradiol. The slope of the line indicates the magnitude of the causal relationship. (B) Funnel plot. (C) Forest plot of MR analysis representing causal estimates of menarche on estradiol. The circles next to each SNP indicate causal estimates for each IV, respectively, and the lowest two circles show multiple-instrument MR analysis using Egger regression and inverse-variance weighted methods. Horizontal lines denote 95% CIs. (D) LOO analyses. The black dots represent one IVW and the red dots represent estimates using all IVs. Horizontal lines indicate 95% confidence intervals. (E) The 1SD value of the age at menarche. Figure S4: 2-sample MR analysis. (A) Scatterplot of the causal effect of vaginitis on menarche. The slope of the line indicates the magnitude of the causal relationship. (B) Forest plot of MR analysis representing causal estimates of vaginitis on menarche. The circles next to each SNP indicate causal estimates for each IV, respectively, and the lowest two circles show multiple-instrument MR analysis using Egger regression and inverse-variance weighted methods. Horizontal lines denote 95% CIs. (C) LOO analyses. The black dots represent one IVW and the red dots represent estimates using all IVs. Horizontal lines indicate 95% confidence intervals. Figure S5: 2-sample MR analysis. (A) Scatterplot of the causal effect of menarche on estradiol. The slope of the line indicates the magnitude of the causal relationship. (B) Forest plot of MR analysis representing causal estimates of menarche on estradiol. The circles next to each SNP indicate causal estimates for each IV, respectively, and the lowest two circles show multiple-instrument MR analysis using Egger regression and inverse-variance weighted methods. Horizontal lines denote 95% CIs. (C) LOO analyses. The black dots represent one IVW and the red dots represent estimates using all IVs. Horizontal lines indicate 95% confidence intervals. Figure S6: 2-sample MR analysis. (A) Scatterplot of the causal effect of age at menopause on vaginitis. The slope of the line indicates the magnitude of the causal relationship. (B) Forest plot of MR analysis representing causal estimates of age at menopause on vaginitis. The circles next to each SNP indicate causal estimates for each IV, respectively, and the lowest two circles show multiple-instrument MR analysis using Egger regression and inverse-variance weighted methods. Horizontal lines denote 95% CIs. (C) LOO analyses. The black dots represent one IVW and the red dots represent estimates using all IVs. Horizontal lines indicate 95% confidence intervals. Figure S7: 2-sample MR analysis. (A) Scatterplot of the causal effect of vaginitis on age at menopause. The slope of the line indicates the magnitude of the causal relationship. (B) Forest plot of MR analysis representing causal estimates of vaginitis on age at menopause. The circles next to each SNP indicate causal estimates for each IV, respectively, and the lowest two circles show multiple-instrument MR analysis using Egger regression and inverse-variance weighted methods. Horizontal lines denote 95% CIs. (C) LOO analyses. The black dots represent one IVW and the red dots represent estimates using all IVs. Horizontal lines indicate 95% confidence intervals.

Supplementary Material 2.

Supplementary Material 3.

## Data Availability

The datasets used and/or analyzed during the present study are available from the corresponding author on reasonable request.
