# Supplementary material for: Strong evidence supports the use of estradiol therapy for the treatment of vaginal inflammation: a two-way Mendelian randomization study
Source: Eur J Med Res. 2024 Jun 18;29:339. doi: 10.1186/s40001-024-01914-4 (PMC11186076; doi:10.1186/s40001-024-01914-4)
Supplement: Supplementary file 1 — Supplementary Material 1. Figure S1: 2-sample MR analysis. (A) Scatterplot of the causal effect of estradiol on vaginitis. The slope of the line indicates the magnitude of the causal relationship. (B) Forest plot of MR analysis representing causal estimates of estradiol on vaginitis. The circles next to each SNP indicate causal estimates for each IV, respectively, and the lowest two circles show multiple-instrument MR analysis using Egger regression and inverse-variance weighted methods. Horizontal lines denote 95% CIs. (C) LOO analyses. The black dots represent one IVW and the red dots represent estimates using all IVs. Horizontal lines indicate 95% confidence intervals. Figure S2: 2-sample MR analysis. (A) Scatterplot of the causal effect of vaginitis on estradiol. The slope of the line indicates the magnitude of the causal relationship. (B) Forest plot of MR analysis representing causal estimates of vaginitis on estradiol. The circles next to each SNP indicate causal estimates for each IV, respectively, and the lowest two circles show multiple-instrument MR analysis using Egger regression and inverse-variance weighted methods. Horizontal lines denote 95% CIs. (C) LOO analyses. The black dots represent one IVW and the red dots represent estimates using all IVs. Horizontal lines indicate 95% confidence intervals. Figure S3: 2-sample MR analysis. (A) Scatterplot of the causal effect of menarche on estradiol. The slope of the line indicates the magnitude of the causal relationship. (B) Funnel plot. (C) Forest plot of MR analysis representing causal estimates of menarche on estradiol. The circles next to each SNP indicate causal estimates for each IV, respectively, and the lowest two circles show multiple-instrument MR analysis using Egger regression and inverse-variance weighted methods. Horizontal lines denote 95% CIs. (D) LOO analyses. The black dots represent one IVW and the red dots represent estimates using all IVs. Horizontal lines indicate 95% confidence inter [file 40001_2024_1914_MOESM1_ESM.docx]

**Supplementary Figures:**

**
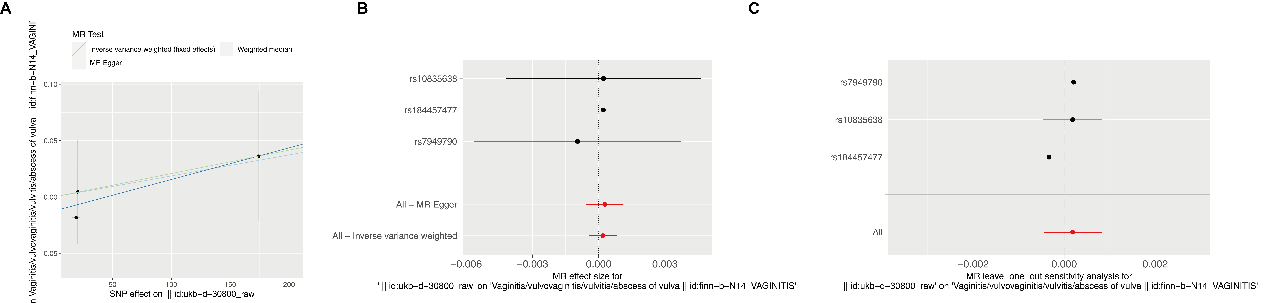
**

**Figure S1:** 2-sample MR analysis. (A) Scatterplot of the causal effect of estradiol on vaginitis. The slope of the line indicates the magnitude of the causal relationship. (B) Forest plot of MR analysis representing causal estimates of estradiol on vaginitis. The circles next to each SNP indicate causal estimates for each IV, respectively, and the lowest two circles show multiple-instrument MR analysis using Egger regression and inverse-variance weighted methods. Horizontal lines denote 95% CIs. (C) LOO analyzes. Per The black dots represent one IVW and the red dots represent estimates using all IVs. Horizontal lines indicate 95% confidence intervals.


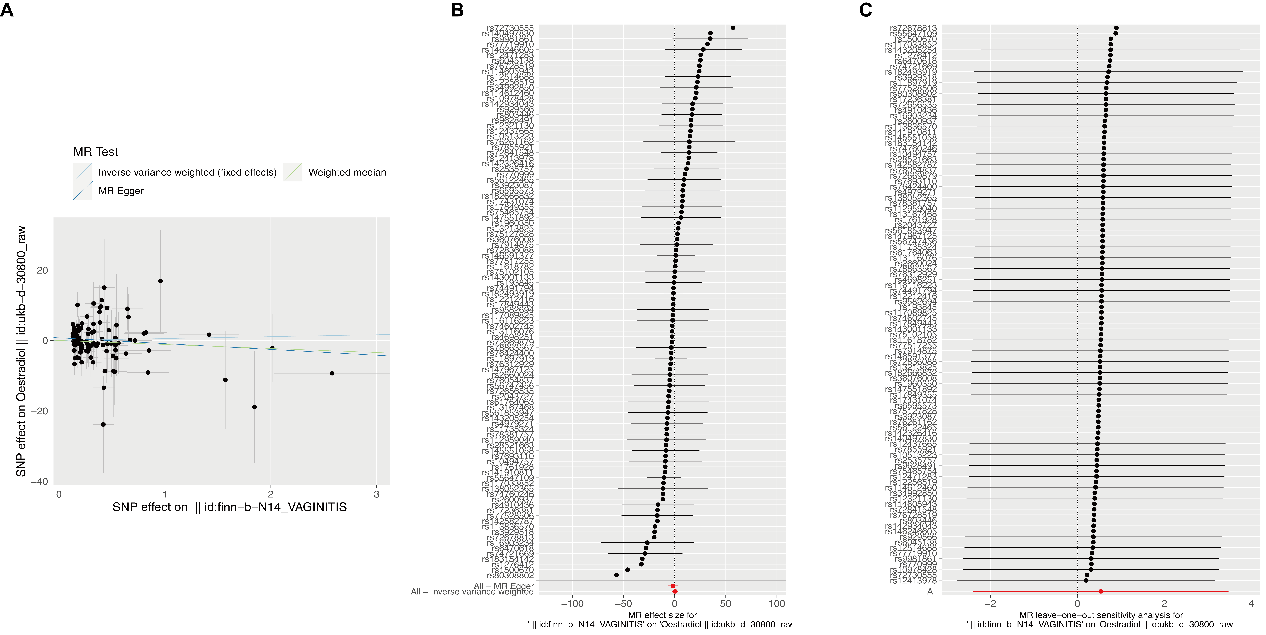


**Figure S2:** 2-sample MR analysis. (A) Scatterplot of the causal effect of vaginitis on estradiol. The slope of the line indicates the magnitude of the causal relationship. (B) Forest plot of MR analysis representing causal estimates of vaginitis on estradiol. The circles next to each SNP indicate causal estimates for each IV, respectively, and the lowest two circles show multiple-instrument MR analysis using Egger regression and inverse-variance weighted methods. Horizontal lines denote 95% CIs. (C) LOO analyzes. Per The black dots represent one IVW and the red dots represent estimates using all IVs. Horizontal lines indicate 95% confidence intervals.


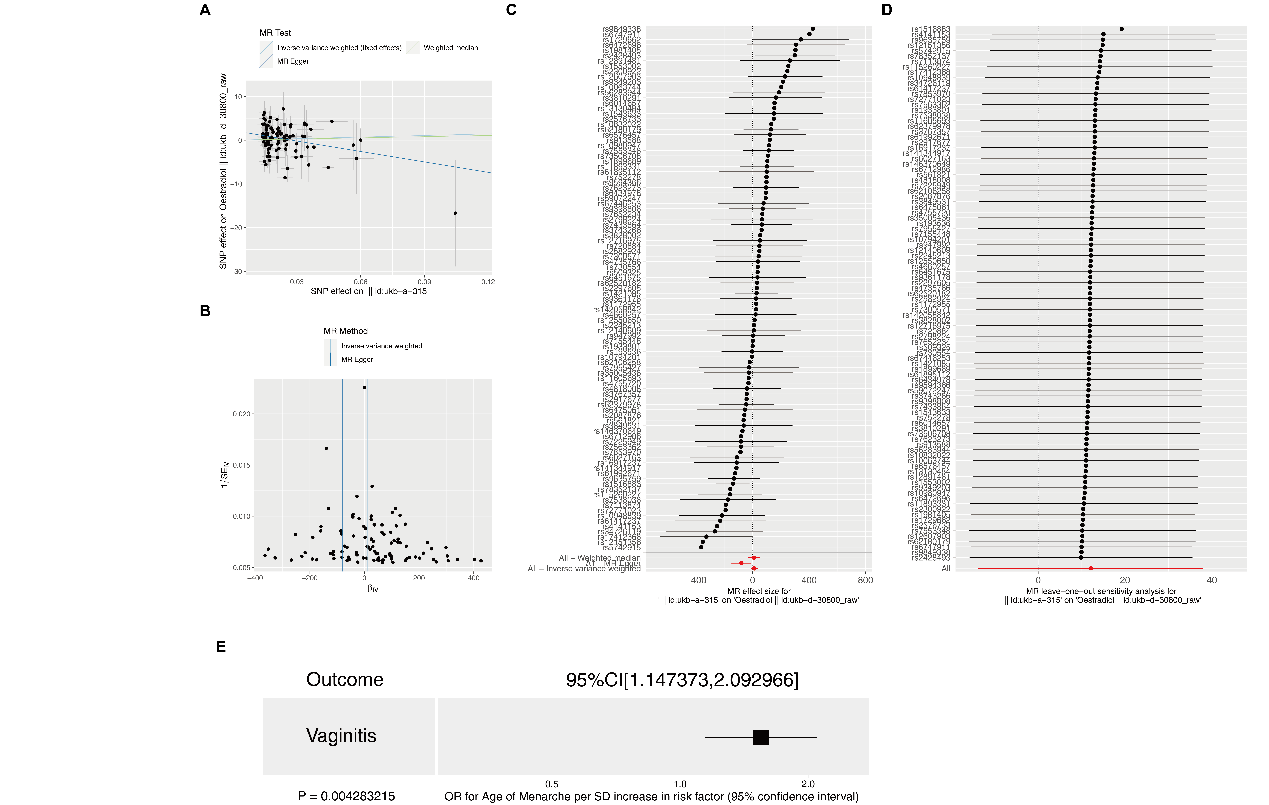


**Figure S3:** 2-sample MR analysis. (A) Scatterplot of the causal effect of menarche on estradiol. The slope of the line indicates the magnitude of the causal relationship. (B) Funnel plot. (C) Forest plot of MR analysis representing causal estimates of menarche on estradiol. The circles next to each SNP indicate causal estimates for each IV, respectively, and the lowest two circles show multiple-instrument MR analysis using Egger regression and inverse-variance weighted methods. Horizontal lines denote 95% CIs. (D) LOO analyzes. Per The black dots represent one IVW and the red dots represent estimates using all IVs. Horizontal lines indicate 95% confidence intervals. (E) The 1SD value of the age at menarche.


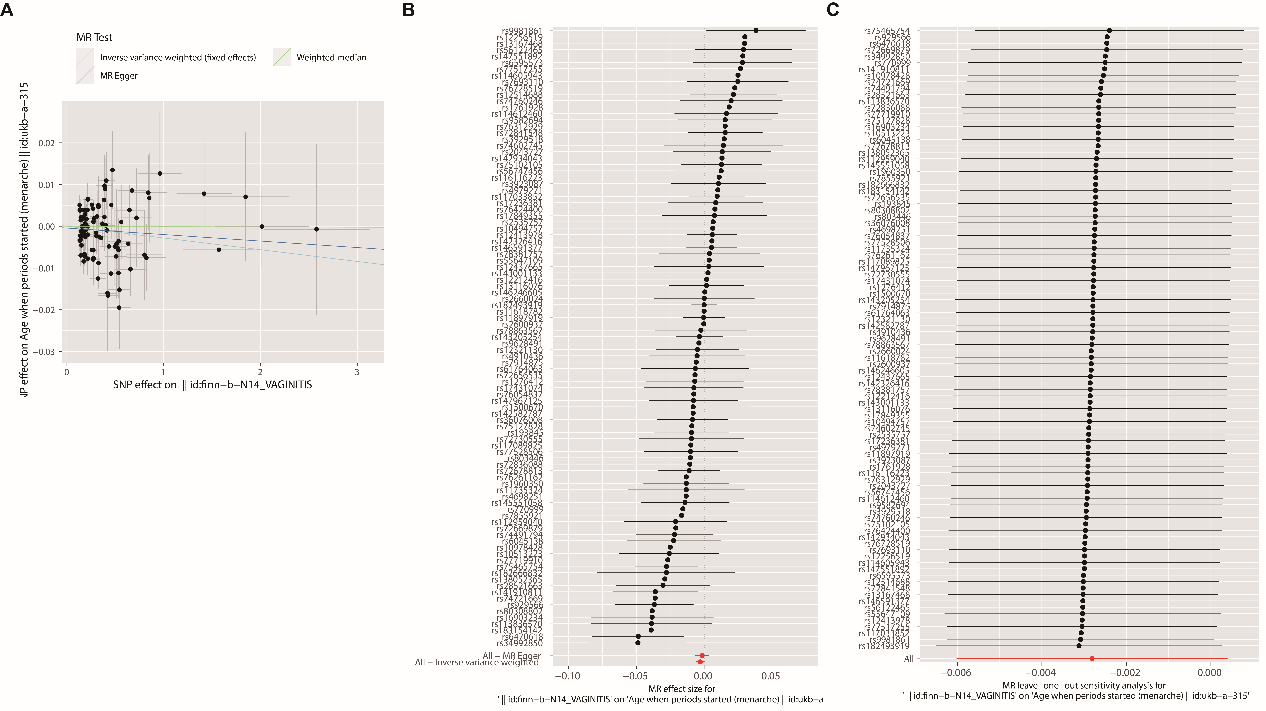


**Figure S4:** 2-sample MR analysis. (A) Scatterplot of the causal effect of vaginitis on menarche. The slope of the line indicates the magnitude of the causal relationship. (B) Forest plot of MR analysis representing causal estimates of vaginitis on menarche. The circles next to each SNP indicate causal estimates for each IV, respectively, and the lowest two circles show multiple-instrument MR analysis using Egger regression and inverse-variance weighted methods. Horizontal lines denote 95% CIs. (C) LOO analyzes. Per The black dots represent one IVW and the red dots represent estimates using all IVs. Horizontal lines indicate 95% confidence intervals.


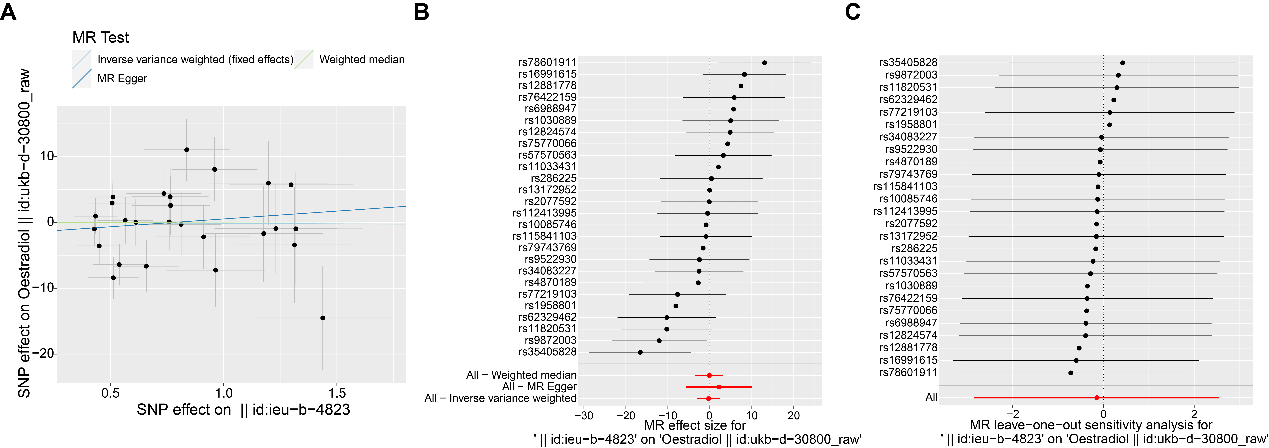


**Figure S5:** 2-sample MR analysis. (A) Scatterplot of the causal effect of menarche on estradiol. The slope of the line indicates the magnitude of the causal relationship. (B) Forest plot of MR analysis representing causal estimates of menarche on estradiol. The circles next to each SNP indicate causal estimates for each IV, respectively, and the lowest two circles show multiple-instrument MR analysis using Egger regression and inverse-variance weighted methods. Horizontal lines denote 95% CIs. (C) LOO analyzes. Per The black dots represent one IVW and the red dots represent estimates using all IVs. Horizontal lines indicate 95% confidence intervals.


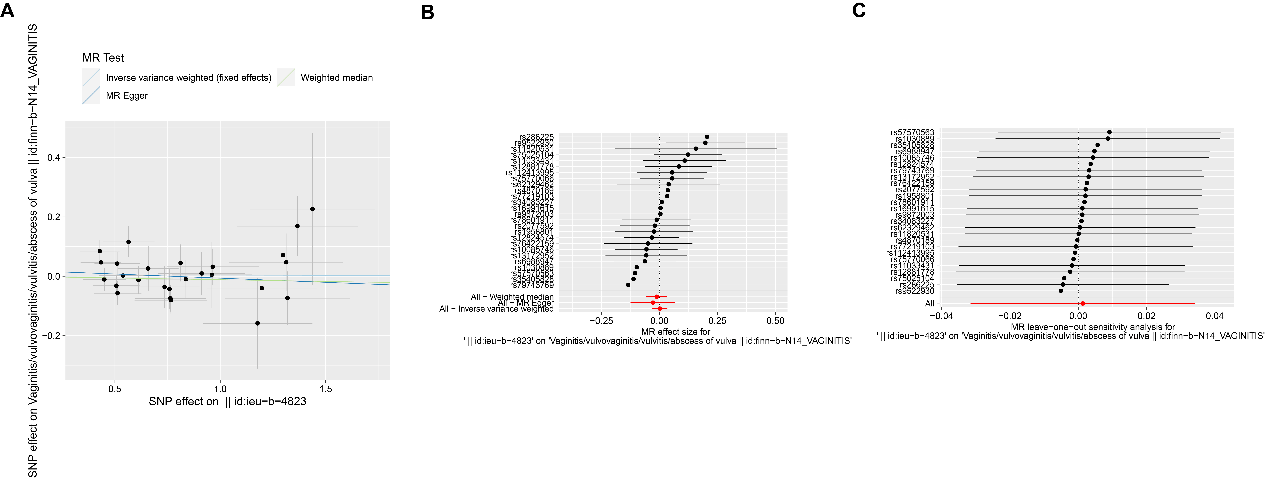


**Figure S6:** 2-sample MR analysis. (A) Scatterplot of the causal effect of age at menopause on vaginitis. The slope of the line indicates the magnitude of the causal relationship. (B) Forest plot of MR analysis representing causal estimates of age at menopause on vaginitis. The circles next to each SNP indicate causal estimates for each IV, respectively, and the lowest two circles show multiple-instrument MR analysis using Egger regression and inverse-variance weighted methods. Horizontal lines denote 95% CIs. (C) LOO analyzes. Per The black dots represent one IVW and the red dots represent estimates using all IVs. Horizontal lines indicate 95% confidence intervals.


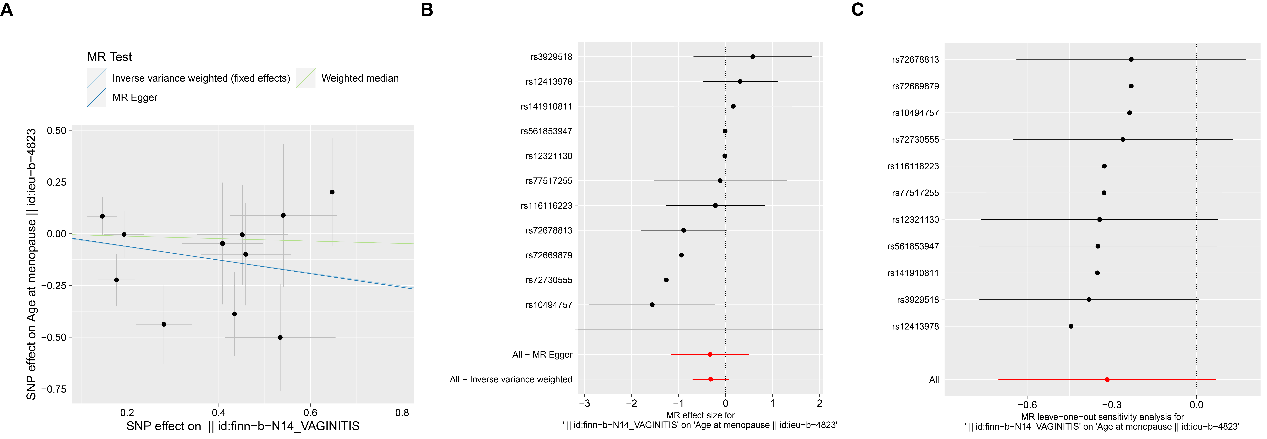


**Figure S7:** 2-sample MR analysis. (A) Scatterplot of the causal effect of vaginitis on age at menopause. The slope of the line indicates the magnitude of the causal relationship. (B) Forest plot of MR analysis representing causal estimates of vaginitis on age at menopause. The circles next to each SNP indicate causal estimates for each IV, respectively, and the lowest two circles show multiple-instrument MR analysis using Egger regression and inverse-variance weighted methods. Horizontal lines denote 95% CIs. (C) LOO analyzes. Per The black dots represent one IVW and the red dots represent estimates using all IVs. Horizontal lines indicate 95% confidence intervals.
