# Supplementary material for: Strong evidence supports the use of estradiol therapy for the treatment of vaginal inflammation: a two-way Mendelian randomization study
Source: Eur J Med Res. 2024 Jun 18;29:339. doi: 10.1186/s40001-024-01914-4 (PMC11186076; doi:10.1186/s40001-024-01914-4)
Supplement: Supplementary file 3 — Supplementary Material 3. [file 40001_2024_1914_MOESM3_ESM.docx]

**Estradiol and HPV16**

| **exposure** | **outcome** | **method** | **nsnp** | **β** | **se** | **pval** | **or** | **or_lci95** | **or_uci95** | **Q** | **Q_pval** |
| --- | --- | --- | --- | --- | --- | --- | --- | --- | --- | --- | --- |
| Estradiol | HPV E7 Type 16 | MR Egger | 26 | -0.0021 | 0.0029 | 0.4883 | 0.9979 | 0.9922 | 1.0037 | 37.2343 | 0.0415 |
|  |  | Weighted median | 26 | -0.0009 | 0.0012 | 0.4450 | 0.9991 | 0.9968 | 1.0014 |  |  |
|  |  | Inverse variance weighted | 26 | -0.0002 | 0.0010 | 0.8379 | 0.9998 | 0.9979 | 1.0017 | 37.9407 | 0.0469 |

**HPV16 and Estradiol**

| **exposure** | **outcome** | **method** | **nsnp** | **β** | **se** | **pval** | **or** | **or_lci95** | **or_uci95** | **Q** | **Q_pval** |
| --- | --- | --- | --- | --- | --- | --- | --- | --- | --- | --- | --- |
| HPV E7 Type 16 | Estradiol | MR Egger | 26 | 3.44259722 | 8.83373086 | 0.70018757 | 31.2680628 | 9.46E-07 | 1034009408 | 35.786919 | 0.05755462 |
|  |  | Weighted median | 26 | -4.0320117 | 3.75386117 | 0.28277913 | 0.01773861 | 1.13E-05 | 27.8144651 |  |  |
|  |  | Inverse variance weighted | 26 | -0.716738 | 3.03564881 | 0.81334963 | 0.48834264 | 0.00127271 | 187.379072 | 36.1633148 | 0.06915812 |

**Estradiol and HPV18**

| **exposure** | **outcome** | **method** | **nsnp** | **β** | **se** | **pval** | **or** | **or_lci95** | **or_uci95** | **Q** | **Q_pval** |
| --- | --- | --- | --- | --- | --- | --- | --- | --- | --- | --- | --- |
| Estradiol | HPV E7 Type18 | MR Egger | 26 | -0.0005909 | 0.00233905 | 0.80271296 | 0.99940928 | 0.99483795 | 1.00400163 | 16.9845462 | 0.8493204 |
|  |  | Weighted median | 26 | -0.0007837 | 0.00109085 | 0.47250616 | 0.99921663 | 0.99708252 | 1.00135531 |  |  |
|  |  | Inverse variance weighted | 26 | -0.0010275 | 0.00077291 | 0.18370104 | 0.99897298 | 0.99746077 | 1.00048748 | 17.0236676 | 0.88093351 |

**HPV18 and Estradiol**

| **exposure** | **outcome** | **method** | **nsnp** | **β** | **se** | **pval** | **or** | **or_lci95** | **or_uci95** | **Q** | **Q_pval** |
| --- | --- | --- | --- | --- | --- | --- | --- | --- | --- | --- | --- |
| HPV E7 Type18 | Estradiol | MR Egger | 12 | -9.1374823 | 14.4518776 | 0.54139617 | 0.00010756 | 5.37E-17 | 215441049 | 8.32675521 | 0.59695313 |
|  |  | Weighted median | 12 | 3.90576649 | 5.1661872 | 0.44963423 | 49.6881508 | 0.00198932 | 1241081.09 |  |  |
|  |  | Inverse variance weighted | 12 | 4.35695764 | 3.84785902 | 0.25750539 | 78.0194099 | 0.04138462 | 147084.321 | 9.26516906 | 0.59743052 |

**Vaginitis and HPV16**

| **exposure** | **outcome** | **method** | **nsnp** | **β** | **se** | **pval** | **or** | **or_lci95** | **or_uci95** | **Q** | **Q_pval** |
| --- | --- | --- | --- | --- | --- | --- | --- | --- | --- | --- | --- |
| Vaginitis | HPV E7 Type 16 | MR Egger | 26 | -0.0717598 | 0.1468918 | 0.62961443 | 0.9307544 | 0.69790816 | 1.24128619 | 23.5903571 | 0.48521062 |
|  |  | Weighted median | 26 | -0.0863826 | 0.08528619 | 0.31112898 | 0.91724321 | 0.77604595 | 1.08413053 |  |  |
|  |  | Inverse variance weighted | 26 | -0.0732884 | 0.05883922 | 0.21292199 | 0.92933276 | 0.82810669 | 1.04293249 | 23.5904861 | 0.54312417 |

**HPV16 and Vaginitis**

| **exposure** | **outcome** | **method** | **nsnp** | **β** | **se** | **pval** | **or** | **or_lci95** | **or_uci95** | **Q** | **Q_pval** |
| --- | --- | --- | --- | --- | --- | --- | --- | --- | --- | --- | --- |
| HPV E7 Type 16 | Vaginitis | MR Egger | 26 | 0.15004946 | 0.10470289 | 0.16472901 | 1.16189171 | 0.94632596 | 1.42656167 | 29.6426608 | 0.19687881 |
|  |  | Weighted median | 26 | 0.03436001 | 0.04598016 | 0.4548951 | 1.03495713 | 0.94576506 | 1.13256063 |  |  |
|  |  | Inverse variance weighted | 26 | -0.0048848 | 0.03684394 | 0.89452554 | 0.99512714 | 0.9257983 | 1.0696477 | 32.70517 | 0.13860498 |

**Vaginitis and HPV18**

| **exposure** | **outcome** | **method** | **nsnp** | **β** | **se** | **pval** | **or** | **or_lci95** | **or_uci95** | **Q** | **Q_pval** |
| --- | --- | --- | --- | --- | --- | --- | --- | --- | --- | --- | --- |
| Vaginitis | HPV E7 Type18 | MR Egger | 26 | 0.02764694 | 0.14659536 | 0.85199705 | 1.02803266 | 0.77129838 | 1.37022348 | 21.6729711 | 0.59877884 |
|  |  | Weighted median | 26 | 0.00217611 | 0.08700176 | 0.98004515 | 1.00217848 | 0.84506026 | 1.18850899 |  |  |
|  |  | Inverse variance weighted | 26 | 0.06792886 | 0.05873812 | 0.24748909 | 1.07028916 | 0.95389864 | 1.20088115 | 21.7629173 | 0.64939477 |

**HPV18 and Vaginitis**

| **exposure** | **outcome** | **method** | **nsnp** | **β** | **se** | **pval** | **or** | **or_lci95** | **or_uci95** | **Q** | **Q_pval** |
| --- | --- | --- | --- | --- | --- | --- | --- | --- | --- | --- | --- |
| HPV E7 Type18 | Vaginitis | MR Egger | 12 | -0.0739305 | 0.16970603 | 0.67235224 | 0.92873626 | 0.66594094 | 1.29523653 | 10.130917 | 0.4290836 |
|  |  | Weighted median | 12 | 0.08454474 | 0.0662753 | 0.20207577 | 1.08822153 | 0.95565831 | 1.23917313 |  |  |
|  |  | Inverse variance weighted | 12 | 0.04251032 | 0.04774243 | 0.37324603 | 1.04342682 | 0.95021705 | 1.14577984 | 10.6494322 | 0.4730826 |

**Estradiol and Lactobacillus**

| **exposure** | **outcome** | **method** | **nsnp** | **β** | **se** | **pval** | **or** | **or_lci95** | **or_uci95** | **Q** | **Q_pval** |
| --- | --- | --- | --- | --- | --- | --- | --- | --- | --- | --- | --- |
| Estradiol | genus Lactobacillus id.1837 | MR Egger | 63 | 0.00126768 | 0.0008484 | 0.14027525 | 1.00126849 | 0.99960491 | 1.00293484 | 65.2076305 | 0.33265848 |
|  |  | Weighted median | 63 | 0.00012845 | 0.00028768 | 0.65523492 | 1.00012846 | 0.99956469 | 1.00069255 |  |  |
|  |  | Inverse variance weighted | 63 | -2.09E-05 | 0.00020684 | 0.91938491 | 0.99997907 | 0.99957375 | 1.00038455 | 67.8258552 | 0.28532517 |

**Lactobacillus and Estradiol**

| **exposure** | **outcome** | **method** | **nsnp** | **β** | **se** | **pval** | **or** | **or_lci95** | **or_uci95** | **Q** | **Q_pval** |
| --- | --- | --- | --- | --- | --- | --- | --- | --- | --- | --- | --- |
| genus Lactobacillus id.1837 | Estradiol | MR Egger | 46 | -22.605286 | 14.2037361 | 0.11865641 | 1.52E-10 | 1.24E-22 | 187.548378 | 41.1887673 | 0.59281127 |
|  |  | Weighted median | 46 | -3.9974257 | 7.03084905 | 0.56965805 | 0.01836285 | 1.90E-08 | 17730.4442 |  |  |
|  |  | Inverse variance weighted | 46 | -2.239781 | 4.82955179 | 0.64281553 | 0.10648182 | 8.25E-06 | 1374.90574 | 43.5133396 | 0.53503249 |

**Vaginitis and Lactobacillus**

| **exposure** | **outcome** | **method** | **nsnp** | **β** | **se** | **pval** | **or** | **or_lci95** | **or_uci95** | **Q** | **Q_pval** |
| --- | --- | --- | --- | --- | --- | --- | --- | --- | --- | --- | --- |
| Vaginitis | genus Lactobacillus id.1837 | MR Egger | 45 | -0.1051673 | 0.06882917 | 0.13384894 | 0.90017387 | 0.78657082 | 1.03018441 | 31.9502303 | 0.89226429 |
|  |  | Weighted median | 45 | -0.0135088 | 0.02649431 | 0.61013908 | 0.98658205 | 0.93665746 | 1.03916766 |  |  |
|  |  | Inverse variance weighted | 45 | -0.0265108 | 0.01811145 | 0.14325942 | 0.9738375 | 0.93987417 | 1.00902813 | 33.353325 | 0.87887524 |

**Lactobacillus and Vaginitis**

| **exposure** | **outcome** | **method** | **nsnp** | **β** | **se** | **pval** | **or** | **or_lci95** | **or_uci95** | **Q** | **Q_pval** |
| --- | --- | --- | --- | --- | --- | --- | --- | --- | --- | --- | --- |
| genus Lactobacillus id.1837 | Vaginitis | MR Egger | 45 | -0.0197998 | 0.17657013 | 0.91123776 | 0.98039494 | 0.69358793 | 1.38580011 | 41.4157715 | 0.54014009 |
|  |  | Weighted median | 45 | -0.0557692 | 0.09247963 | 0.54648008 | 0.9457574 | 0.7889682 | 1.13370483 |  |  |
|  |  | Inverse variance weighted | 45 | 0.01646388 | 0.06277742 | 0.79312248 | 1.01660016 | 0.89890327 | 1.14970757 | 41.4640552 | 0.58090895 |
